# Impacts of household water demands and water heater delivery temperatures on opportunistic premise plumbing pathogens (OPPPs) in a residential setting

**DOI:** 10.1016/j.temicr.2025.100002

**Published:** 2025-03

**Authors:** Alshae’ Logan-Jackson, Jumana Alja’fari, Visesh Uppoor, Tania Ullah, Jennifer Dootz

**Affiliations:** a Building Energy and Environment Division, Heat Transfer and Alternative Energy Systems Group, National Institute of Standards and Technology, 100 Bureau Drive, Gaithersburg, MD 20899, USA; b Biosystems and Biomaterials Division, Complex Microbial Systems Group, National Institute of Standards and Technology, 100 Bureau Drive, Gaithersburg, MD 20899, USA; c Biosystems and Biomaterials Division, Microbial System Focus Group, National Institute of Standards and Technology, 100 Bureau Drive, Gaithersburg, MD 20899, USA; d The University of Maryland, College Park, MD, 20742, USA

**Keywords:** Household water demands, Water heater temperature, Opportunistic premise plumbing pathogens (OPPPs), Legionella pneumophila, PEX manifold

## Abstract

Efforts aimed towards water and energy conservation within residential buildings may create enhanced conditions for Opportunistic Premise Plumbing Pathogens (OPPPs) growth. To investigate this issue, a study was conducted to assess the effect of water demands and water temperature on four OPPPs – *L. pneumophila, P. aeruginosa, M. avium,* and *N. fowleri* – in a residential test facility. Two water delivery temperatures (54 and 66 °C) and household water demands (low: 47.3 or 127.9 L; high: 263.1 L) were evaluated. A total of 260 samples were collected and analyzed using droplet digital polymerase chain reaction (ddPCR). Samples were collected from the influent water pipe, water heater, PEX manifold, kitchen and bathroom faucets, tub, and shower. The most frequently detected OPPP was *L. pneumophila*, which was found in 8.1 % of water samples, followed by *P. aeruginosa* (3.5 %), and *M. avium* (1.9 %). Water demand and water heater setpoint temperature were independently associated with the detection and concentration of *L. pneumophila*. Kitchen and bathroom faucets posed a higher risk of exposure to *L. pneumophila* compared to the tub and shower. The current dataset represents the largest-scale investigation to date of the effect of household operating conditions on OPPPs. This dataset can be used in quantitative microbial risk assessment (QMRA) models to estimate the potential risk of infection with OPPPs.

## 1. Introduction

The United States Environmental Protection Agency estimates that approximately 70 % of household water demand occurs indoors (U.S. Environmental Protection Agency (U.S.EPA), 2021). The need to reduce water use in buildings has increased in recent years with increasing population and water shortages (U.S. Environmental Protection Agency (U.S.EPA), 2021). Water conservation strategies, such as water conserving fixtures, have decreased water flows and lowered daily water use (U.S. Environmental Protection Agency (U.S.EPA), 2021). While these steps are improving water efficiency, they may have unintended consequences on microbial water quality in buildings (Rhoads et al., 2015; Ji et al., 2017).

Water heating in residential buildings accounts for approximately 20 % of residential energy use, and it is the second largest energy expense in a dwelling (Department of Energy (DOE), 2022). Lowering water heater temperatures to 49 °C (120°F) (Department of Energy (DOE), 2022) is a common recommendation for conserving energy and reducing carbon emissions. Other ways to conserve water heating energy in residential buildings include installing water-efficient fixtures, insulating hot water lines (U.S. Environmental Protection Agency (U.S.EPA), 2017), and using hot and cold manifold water delivery systems with cross-linked polyethylene (PEX) tubing (Rodriguez, 2019). PEX is a plastic pipe material that reduces water use and heat losses (Rodriguez, 2019) because the flexible tubing allows for fewer connections that reduce the chance of leaks, shorter pipe lengths from water supply to the fixtures, and lower heat transfer relative to metal pipe. Manifold systems combined with PEX tubing are approved for use in potable hot and cold water supply systems in all model plumbing and mechanical codes across the United States and Canada (Home Innovation Research Labs, 2013). Therefore, consideration of this configuration is important for assessing water quality in residential buildings. There is currently an incomplete understanding of how water systems and their associated residential plumbing components contribute to the occurrence and concentration of Opportunistic Premise Plumbing Pathogens (OPPPs), and how best to control the associated public health risk in buildings.

Efforts to conserve water have led to lower and less frequent water flows, which can create stagnation of water in pipes (Rhoads et al., 2015; Ji et al., 2017). Pipes with low or infrequent flows could serve as sites for the amplification of water-borne pathogens, such as *Legionella* (Schwake et al., 2016). If efficient fixtures are combined with “right-sized” pipes, these concerns may be reduced (The International Association of Plumbing and Mechanical Officials (IAPMO), 2020). Other reasons why low household water demands may lead to an increased presence of OPPPs are the lack of replenishment of disinfectant residuals which decay over time, the variation in temperatures within pipes that may lead to conditions conducive to growth, and less hydraulic disturbances in pipes that may interfere with pathogen growth environments (Rhoads et al., 2015; Proctor et al., 2017; Donohue et al., 2019). The interaction of water temperature, household water demands, and disinfectant residual, as well as the complexity of plumbing and fixture components, need to be considered holistically to understand the ecology of OPPPs, especially in residential buildings, as they have not been as well characterized and monitored as commercial buildings (Donohue et al., 2019; Buse et al., 2020; Logan-Jackson et al., 2023, 2021). Therefore, an improved understanding is needed on how water and energy conservation efforts play a potential role in the growth of OPPPs in residential buildings.

The extent of exposure and potential risk of infection with OPPPs in single-family residences are not well characterized due to the lack of data on the occurrence and concentration of these pathogens in residential households. A mathematical modeling approach referred to as Quantitative Microbial Risk Assessment (QMRA) can be used to estimate infection and/or illness risk when a population is exposed to pathogenic microorganisms in the environment (Minnesota Department of Health, 2024). Data on the detection rates and concentrations of OPPPs in water samples collected from kitchen faucets, showers, bathroom faucets, and bathtubs under different water demands and water heater temperatures can be used as inputs to QMRA models to estimate infection/illness risks and identify the most significant factors driving these risks.

In this study, we investigated the effects of household water demands and water heater delivery temperature on the occurrence and concentrations of *Legionella pneumophila, Pseudomonas aeruginosa, Mycobacterium avium* and, *Naegleria fowleri* in a residential test facility with water-efficient fixtures and a PEX-manifold system. The methodology and data presented can serve as a basis for expanded investigations of the potential for OPPPs growth in residential water systems. Furthermore, the resulting dataset can be used to develop QMRA models to estimate the risk of infection with OPPPs under risk scenarios specific to residential settings.

## 2. Materials and methods

### 2.1. Description of the National Institute of Standards and Technology Net-Zero Energy Residential Test Facility plumbing

Data were collected from the water heating and plumbing system in the Net-Zero Energy Residential Test Facility (NZERTF), a 252 m^2^ (2 712 ft^2^) research house located at the National Institute of Standards and Technology (NIST) in Gaithersburg, MD, USA. The NZERTF is used to measure performance and document new building technologies and designs for achieving net-zero energy consumption. Details on the design of the home and the monitoring efforts can be found in Fanney et al. (2015) and Davis et al. (2014). The NZERTF receives water from the Potomac and Patuxent Water Filtration Plants (WFPs) which treat water originating from the Potomac and Patuxent Rivers, respectively. Details on the water treatment processes at these plants are provided in the Supporting Information section. The time it took for the water to travel from the utility to the NZERTF was approximately 96 h. Once the water reached the NZERTF, the cold water average chlorine residual was below the detectable limit and 0.58 mg/L during the low- and high-water demand profiles, respectively. The cold water entering the NZERTF was heated by a heat-pump water heater that consisted of a 163 L (43 gal) storage tank (located in the basement) and a detached outdoor unit that utilized CO_2_ as the refrigerant, with water circulating between the two during heating. For the tests described in this paper, the thermostat of the water heater was set to deliver water at two temperatures, 54 °C ± 3 °C (130°F ± 5°F) and 66 °C ± 3 °C (150°F ± 5°F), depending on the experimental profile. Details on the operation and temperature measurements of the water heater are provided in the Supporting Information section.

### 2.2. Description of the operational factors and simulated profiles

To evaluate the potential impact of water demand on the growth of OPPPs in the water system of the house, an automated demand schedule was implemented to simulate a hypothetical household use pattern. In addition, the hot water delivery temperature was adjusted using the built-in thermostatic control of the heat-pump water heater. Sample temperatures were also measured at the time of collection using a thermometer with an accuracy of ± 1 °C. The simulated low- and high-water demand included both cold and/or mixed water uses at the selected fixtures in the house as listed in Table S-1. These fixtures were: a kitchen faucet located on the first floor, and showerhead, bathtub spout and faucet located in the owner’s bathroom on the second floor. A detailed description of the daily water demand and sampling schedules for low- and high-water demands is included in the Supporting Information section (Tables S-2 and S-3, respectively). Although several household water demands occurred throughout the day, not all usage events were sampled for water quality measurements. For the experiments described in this paper, household water demands were limited to fixtures, as the clothes washer and dishwasher were not operated during this period.

The low water demand was intended to simulate household water use by a single occupant for bathroom and kitchen activities before and after going to work and who leaves the house on weekends. The total daily water consumption, including cold and mixed water, was approximately 47.3 L (12.5 gal) on Monday through Friday, except for Wednesday when it was 127.9 L (33.8 gal) when the simulated occupant used the bathtub instead of the shower. The high water demand simulated household water use by a family of four occupants who take daily showers and baths and use bathroom and kitchen faucets several times per day and do not leave home on weekends. The resulting total daily water consumption was 263.1 L (69.5 gal) each day of the week. The low water demand resulted in a smaller total water consumption per day and longer stagnation times when no draws occurred, especially during the weekend (58.5 h). This pattern also involved a one-week stagnation between bathtub uses. The high water demand corresponded to a higher water consumption per day and shorter stagnation times.

Four profiles of combined household water demands and delivery temperatures were implemented to test their impact on OPPPs occurrence and concentration (Table 1). Each profile was operated for three weeks. The first week was an acclimation period to allow the microbial community to adjust to the new conditions. Sampling occurred during the two following weeks. For the Low water Demand/Low water Temperature (LD/LT) and Low water Demand/High water Temperature (LD/HT) profiles, water samples were collected twice per week (Mondays and Wednesdays, n = 30 samples per week), while sampling occurred once per week (Tuesdays) for the High water Demand/Low water Temperature (HD/LT) and High water Demand/High water Temperature (HD/HT) profiles (high water demand, n = 35 samples per week). The decision of sampling each water demand at a different frequency was made to ensure a similar number of weekly samples.

### 2.3. Sample collection

A total of 260 water samples, each having a volume of 0.5 L (0.13 gal), were collected between September 2021 and December 2021 from the influent water pipe that feeds the water heater, the top and bottom of the water heater tank, the bottom valve of the hot PEX manifold, and the tested fixtures. All sample bottles contained 1 % sodium thiosulfate to neutralize the residual chlorine. The influent water pipe, water heater, and PEX manifold system were all located in the basement of the home and were sampled before the automated daily use schedule started, which is described as the first draw water sample(s). Only four fixtures were sampled (Table S-1).

Cold and hot water samples were collected as separate samples to evaluate and compare the microbial water quality of the two systems. Mixed water samples were also collected to evaluate the microbial water quality when hot and cold water are mixed. The authors decided to sample bulk water rather than biofilms to keep the plumbing intact during sampling.

### 2.4. Microbial analysis

#### 2.4.1. Water samples processing and DNA extraction

Three hundred mL (0.3 L) of each water sample was processed by filtering through a 47 mm, 0.45 μm polycarbonate membrane (Whatman, Kent, U.K). Processed sample volume was based on the best practice for collecting before and after water use samples for routine testing (at least 0.25 L) (Centers for Disease Control, 2021). The polycarbonate filter was folded into a 1/8 pie shape with the contents of the filter folded to the inside. The filter was then transferred to a 2.0 mL (0.002 L) polypropylene screw cap tube (VWR, Radnor, PA, USA), which was stored at a temperature of −20 °C (−4°F) to be utilized later for DNA extraction. DNA extraction was performed using a FastSpin DNA Kit (MP Biomedicals, Solon, OH, USA) according to the manufacturer’s instructions.

#### 2.4.2. Molecular analysis of L. pneumophila, P. aeruginosa, M. avium, and N. fowleri

Droplet digital PCR (Bio-Rad Laboratories, Hercules, CA, USA) was performed according to the manufacturer’s instructions to analyze each sample for four OPPPs: *L. pneumophila, P. aeruginosa, M. avium,* and *N. fowleri*. The primers and probes that were used are listed in Table S-4. Duplex reactions were performed for two separate assays: the first assay consisted of *L. pneumophila* and *P. aeruginosa*, and the second assay was comprised of *M. avium* and *N. fowleri.* All primers and probes were ordered from Eurofins Genomics Co., (Louisville, KY, USA). Positive controls using genomic DNA for *L. pneumophila* (ATCC No. 33,152)*, P. aeruginosa* (ATCC No. BAA47)*, M. avium* (ATCC No. BAA-968D-5), and *N. fowleri* (ATCC No. 30174D) were obtained from America Type Culture Collection (ATCC, Manassas, VA, USA) and utilized for assay optimization. Positive controls were run with each respective droplet digital PCR (ddPCR) assay as well as a negative control sample (DNA/RNAase free water). A non-template control, i.e., molecular grade water, was used with each ddPCR plate. Negative and positive controls were used to identify contamination (if any) and the efficiency of the assay (i.e., amplification of all target molecules with each cycle of the PCR within each water sample, if present). As part of the quality control, sample results were only considered for analysis when the reader accepted at least 10 000 or more droplets. For each assay, one ddPCR was run with two biologicals (sample collection replicates), and three technical replicates (same extraction) were tested. Biological and technical replicates were run for each sample to determine if there was significant variation among sampling days and whether the assay results were reproducible, respectively.

The ddPCR assay was performed according to the manufacturer’s instructions. In brief, the reaction mixture consisted of 2X supermix (no dUTP) (Bio-Rad Laboratories, Hercules, CA, USA), 900nM (9^−7^ M) forward and reverse primers, and 250 nM (2.5^−7^ M) for each probe (Eurofins Genomics Co., Lousiville, KY, USA), and up to 330 ng (0.33 ug) of environmental and positive control DNA template, to a final volume of 20 μL (0.00002 L). Twenty microliters of the sample reaction mixtures were loaded into ddPCR 96-well plate (semi-skirted; MFG) (Bio-Rad Laboratories, Hercules, CA, USA). The 96-well plate was then placed into an automated droplet generator (Auto DG: Bio-Rad, Laboratories, CA, USA) for droplet generation. Afterward, the ddPCR plate was sealed with pierceable foil heat seals using a PX1^™^ PCR Plate Sealer (Bio-Rad, Laboratories, CA, USA). The plate was amplified using a Bio-Rad C1000 Touch thermal cycler (Bio-Rad, Laboratories, CA, USA). The cycling protocol was as follows: 95 °C (203°F) for 10 min, followed by 40 cycles of 94 °C (201°F) for 30 sec and 57 °C (134°F) for 1 min with a final 10 min cycle at 98 °C (208°F) for 10 min. After endpoint amplification, droplets were read using a QX200 droplet reader (Bio-Rad QX200^™^ Droplet Digital^™^ PCR System, Hecules, CA, USA) and samples were analyzed using the Quantasoft software package v1.7.4 (Bio-Rad, Laboratories, CA, USA).

### 2.5. Statistical analysis

Statistical analyses were conducted on the collected data for each opportunistic pathogen across the entire study period and different operational periods within the study using Minitab^®^, Minitab^®^ LLC (2023). The collected data was categorized into different groups to investigate factors which potentially influenced the occurrence and concentration of OPPPs. Data were grouped by operational profiles (LD/LT, HD/LT, HD/HT, and LD/HT), fixture type (faucet, tub spout, and shower), sample collection site (influent line, heat-pump water heater, PEX manifold, kitchen, and bathroom), and sample type (cold, mixed, and hot).

Statistical distributions were fitted to concentrations of OPPPs using censored data analysis. Censored data refers to measurements which are not quantified and are only known to be lower or higher than a specific threshold (Helsel, 2012). Multiple censored data analysis techniques were considered for distributional fitting and the estimation of summary statistics including imputation methods, robust regression on order statistics (ROS), Kaplan-Meier/Turnbull, robust maximum likelihood estimation (MLE), and the MLE method.

The MLE was found to be a suitable method to estimate descriptive statistics when a high number of measurements (n > 50) is available, and about 50 – 80 % of these measurements are censored (Helsel, 2012). For most data groupings in this study, the number of measurements were greater than 50. As a result, the MLE method within the Reliability/Survival package in Minitab^®^ was used for distributional fitting and estimating descriptive statistics. However, the percentage of censored measurements was always greater than 80 % regardless of the OPPP or data grouping in question. The use of the MLE method or other methods to obtain estimates of descriptive statistics is limited by the high percentage of censoring; nonetheless, for purposes such as hypothesis testing, signals can still be identified at censoring levels near 80 % (Helsel, 2012). Details on the use of the MLE method are provided in the supporting information section.

Censored regression employing the MLE method within Minitab^®^’s Reliability/Survival package was used to find whether concentrations of OPPPs differed significantly between group pairings based on operational profiles, fixture type, collection sites, and sample types. To investigate whether the occurrence of OPPPs differed significantly based on the same group pairings, Chi-square and cross tabulation analysis was used. In cases where tabular cell counts in contingency tables were less than 5, Fisher’s exact test was used instead of Chi-square. Significance of all statistical differences was assumed at a confidence level of 90 % (α-value = 0.1). A 90 % confidence level was chosen because of the low detection of OPPPs, and the uncertainty associated with microbial observations in general. This confidence level has been applied in other studies investigating the presence and concentrations of potentially human-infectious pathogens in roof runoff (Alja’fari et al., 2022) and the presence of fecal indicator bacteria in piped water systems (Taylor et al., 2018).

## 3. Results and discussion

### 3.1. Characterization of OPPPs across the entire study period

*L. pneumophila* was the most frequently detected pathogen of all the investigated OPPPs, while *N. fowleri* was not found in any of the analyzed samples. *L. pneumophila* was detected in 8.1 % (21/260) of the collected samples at concentrations ranging from 3.40 to 4.98 Log_10_ GC/100 mL (Table 2). *P. aeruginosa* was detected in 3.5 % (9/260) of the analyzed samples at concentrations ranging from 3.48 to 5.34 Log_10_ GC/100 mL, and *M. avium* was detected in 1.9 % (5/260) of the collected samples at a concentration range of 3.40 to 3.99 Log_10_ GC/100 mL (Table 2). At a similar retrofitted net-zero energy, water, and waste house, (Ley et al., 2020) did not find *L. pneumophila* in any of the collected samples. However, they found *M. avium* in approximately 1 % (2/259) of the analyzed samples.

The % detection and concentration range of *L. pneumophila* in this study are higher than those reported by Gleason et al. (2023) for a large-scale residential study in New Jersey (Table 3). Gleason et al. (2023) collected 451 samples from the hot water side of showers, hot tubs, and infrequently used taps from 94 households of which 37 had Legionnaires-Disease case patients. The discrepancy in detection and concentration range between the studies could be attributed to the different setting (small versus large scale), sample types, sampling sites, and analytical method applied in this study (ddPCR) which measures both viable and non-viable *L. pneumophila* whereas Gleason et al. (2023) used culture methods which only account for viable *L. pneumophila*.

Wang et al. (2012) reported the occurrence of *L. pneumophila* in 4.4 % of samples (4/90 samples) collected from taps and water heaters in 29 residential households in southwest Virginia at an average concentration of 2.99 Log_10_ (GC/100 mL) for positive samples, which falls below the reported range for positive samples in this study (Table 3). Wang et al. (2012) also investigated *M. avium* and *P. aeruginosa* which were detected in 8.9 % and 1.1 % of the collected samples at average concentrations of 2.04 and 2.26 Log_10_ (GC/100 mL), respectively. These averages fall below the range for *M. avium* and *P. aeruginosa* reported in this study potentially due to different limits of detection.

In a study conducted on *Legionella* infection risk at homes in different Italian regions, Borella et al. (2004) reported *L. pneumophila* counts of 1.1 to 3.54 Log_10_ (CFU/100 mL) which are 1 to 2 orders of magnitude lower than the ones reported in this study (Table 3). This could be due to the different limit of detection, scale of the study, type of collected samples, sampled sites, and the analytical method (molecular versus culture).

The lognormal distribution was found to provide the best fit for *L. pneumophila* (Fig. S-2), *P. aeruginosa* (Fig. S-3), and *M. avium* concentration data (Fig. S-4) across the entire study period. Due to the high percentage of censored data, nearly all the descriptive statistics of OPPPs determined using the MLE method fall below the analytical limit of detection (Fig. S-5). Nevertheless, using the MLE method for samples collected from the kitchen and bathroom faucets, shower, and tub separately from the influent water pipe, heat-pump water heater, and manifold remains useful for comparing observed exposure concentrations with risk-based critical concentrations for indoor residential uses.

**Fig. 1.**
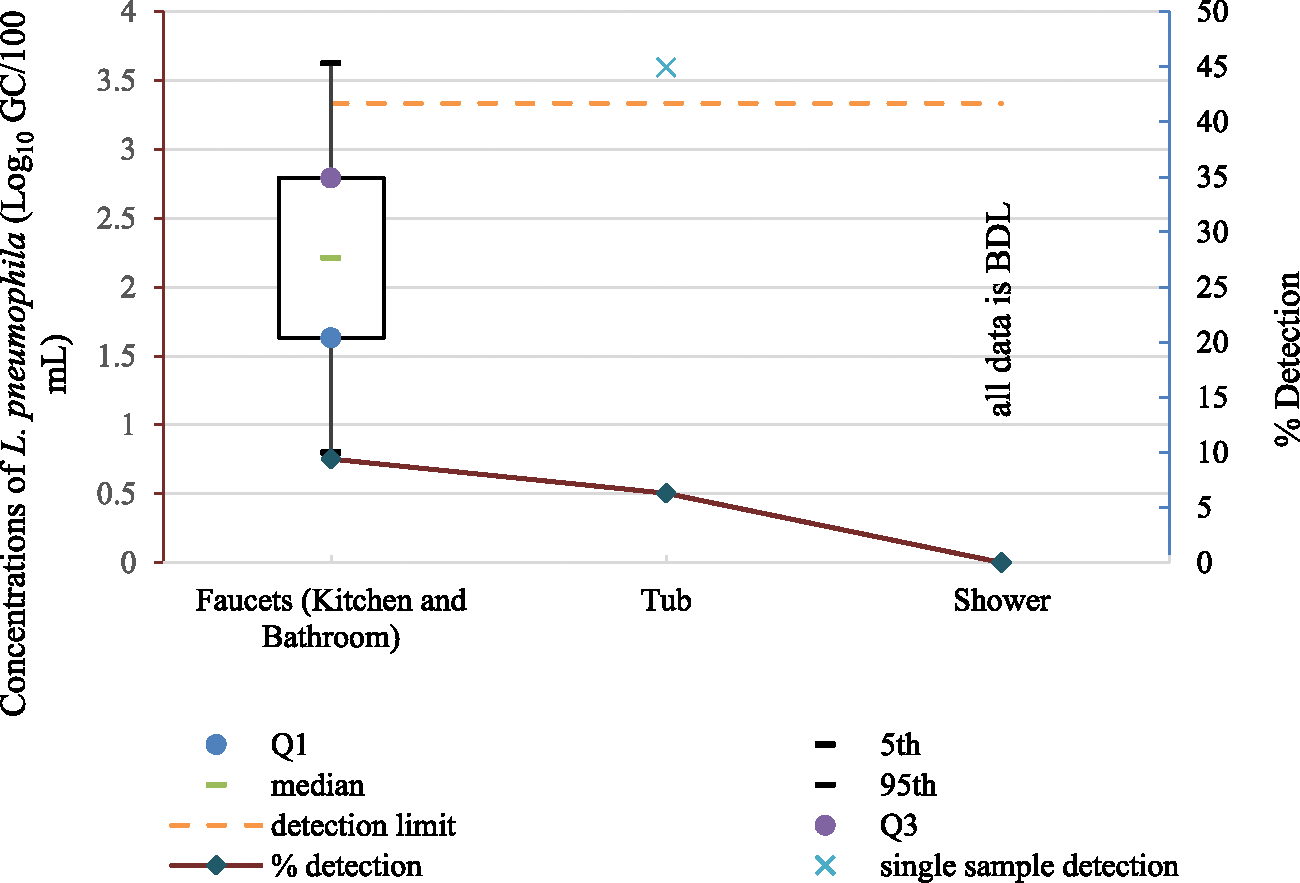
Detection and concentrations of *L. pneumophila* in samples collected from fixtures across the study period (a lognormal distribution was fitted to faucet samples; n_faucet_ = 160, n_tub_ = 16, and n_shower_ = 24).

*L. pneumophila* was found in 9.4 % (15/160) of the samples collected from kitchen and bathroom faucets and 6.3 % (1/16) of the samples collected from the tub (Fig. 1). *L. pneumophila* was not detected in any of the shower samples. The lognormal distribution provided a good fit to *L. pneumophila* concentrations in samples collected from the kitchen and bathroom faucets (Fig. S-6). The high percentage of censored data for *L. pneumophila* in faucet samples limits the effectiveness of the MLE method; however, Helsel (2012) noted that estimates of the 95th percentile can still be obtained for large datasets.

The 95th percentile *L. pneumophila* concentration in faucet samples is two orders of magnitude higher than the 95th percentile infection critical concentration in conventional faucets for a 10^−6^ Disability Adjusted Life Years (DALY) annual risk target calculated by Hamilton et al. (2019) (Table 4). Additionally, the 5th percentile and median *L. pneumophila* concentrations in faucet samples are higher than the 5th percentile and median infection critical concentrations in conventional faucets calculated by Hamilton et al. (2019) for both the 10^−4^ annual and 10^−6^ DALY annual risk targets (Table 4). The infection-critical concentrations reported by Hamilton et al. (2019) were determined for culture-based values whereas concentrations observed in this study were obtained using ddPCR and thus encompass both viable and non-viable *L. pneumophila* cells. Nonetheless, faucets appear to pose a potential risk of exposure to *L. pneumophila* under the current conditions of this study.

### 3.2. Characterization of OPPPs during each operational profile

#### 3.2.1. L. pneumophila

**Fig. 2.**
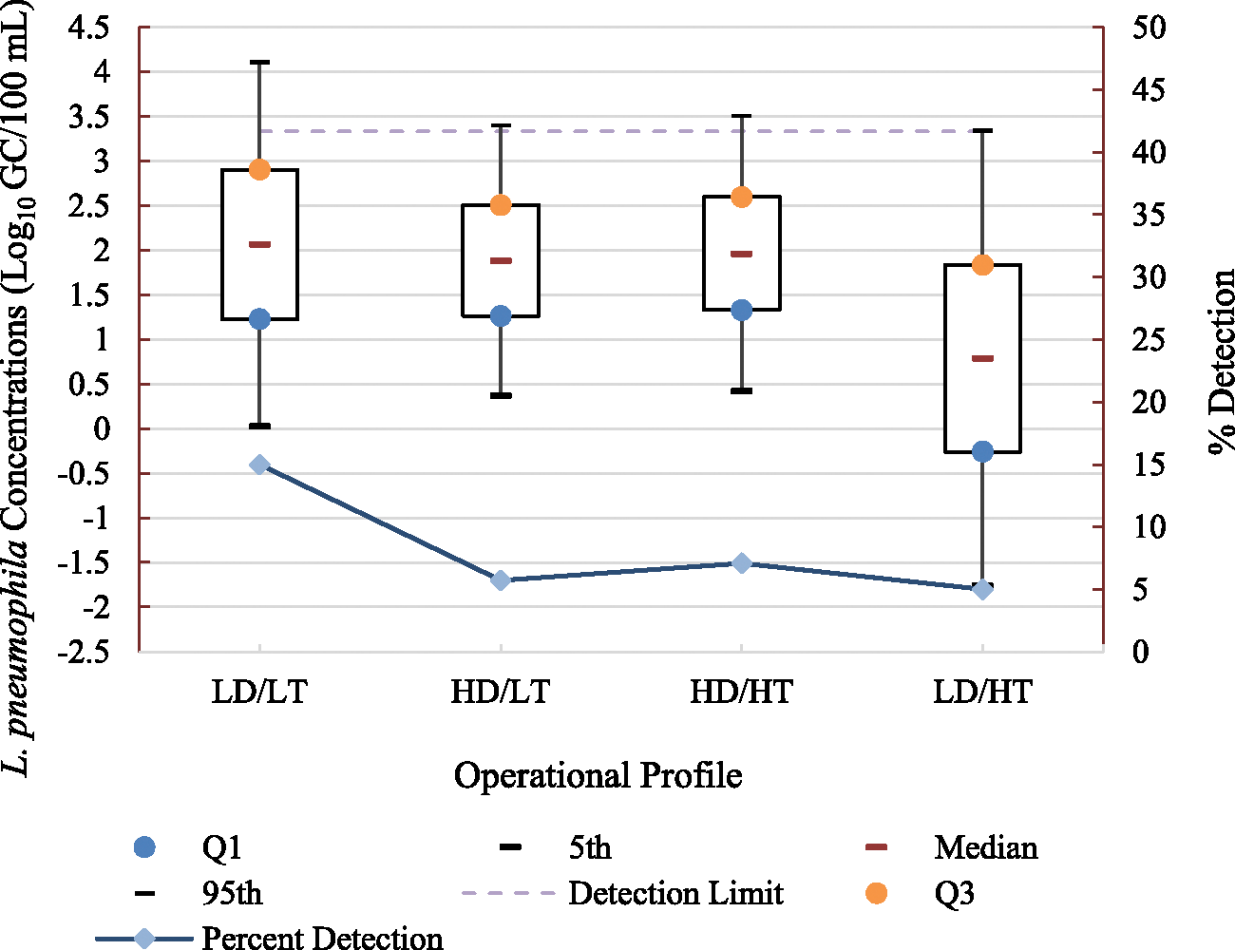
Boxplots of lognormal distribution Fit to *L. pneumophila* concentrations during each operational profile (n_LD/LT_ = 60, n_HD/LT_ = 70, n_HD/HT_ = 70, and n_LD/HT_ = 60).

During the LD/LT profile, *L. pneumophila* was detected in 15 % of the samples at concentrations ranging from 3.60 to 4.98 Log_10_ GC/100 mL (Table 2). A lognormal distribution was found to provide the best fit to *L. pneumophila* data including both censored and uncensored observations (median = 2.07 Log_10_ GC/100 mL and 95th percentile = 4.11 Log_10_ GC/100 mL, Fig. 2 and Fig. S-7). For the HD/LT profile, *L. pneumophila* occurred at a lower rate, i.e., 5.7 % and lower concentrations ranging from 3.40 to 3.95 Log_10_ GC/100 mL compared to the LD/LT profile (Table 2). The lognormal distribution provided a good fit to both censored and uncensored *L. pneumophila* measurements in the HD/LT profile (median = 1.89 Log_10_ GC/100 mL and 95th percentile = 3.40 Log_10_ GC/100 mL, Fig. 2 and Fig. S-8).

The use of the MLE method to obtain estimates of descriptive statistics is limited by the high percentage of censored data across different operational profiles, sampling sites, and sample types; however, for purposes such as regression, correlation, and hypothesis testing, signals can still be identified at censoring levels near 80 % (Helsel, 2012). Using the MLE method to detect differences between profile pairings, the concentrations and % detection of *L. pneumophila* dropped significantly during the HD/LT profile compared to the LD/LT profile (p-values = 0.07 and 0.08, respectively, Table 5). Consequently, increasing water usage while maintaining the heat-pump water heater at a lower setpoint (54 °C ± 3 °C) appeared to result in significant reductions in the detection and concentration of *L. pneumophila*.

To the authors’ knowledge, the effects of water demand and water heater temperature on the concentrations of *L. pneumophila* were not investigated for residential settings (Table 3). However, Ley et al. (2020) reported an indirect relationship between water usage and *Legionella* spp. concentrations at retrofitted net-zero energy, water, and waste house. Ley et al. (2020) reported that lower flows and water use resulted in increased stagnation which in turn was correlated with higher *Legionella* spp. concentrations. Additionally, in a lab-scale study, Rhoads et al. (2015) found that operating an electric household water heater at simulated medium and high-water use frequencies while maintaining the heater’s temperature at 51 °C resulted in a decrease in *L. pneumophila* concentrations at distal taps by two orders of magnitude compared to simulated low use frequency.

When the operational conditions were changed from HD/LT to HD/HT, *L. pneumophila* occurred at a rate of 7.1 % and a concentration range of 3.40 to 4.17 Log_10_ GC/100 mL (Table 1). Censored and uncensored *L. pneumophila* measurements were lognormally distributed (median = 1.96 Log_10_ GC/100 mL and 95th percentile = 3.51 Log_10_ GC/100 mL, Fig. 2 and Fig. S-9). While the concentration range and detection rate of *L. pneumophila* during the HD/HT profile were lower than those measured during the LD/LT profile, these reductions were not statistically significant (p-values = 0.12 and 0.15, respectively, Table 5) even though an increase in both the water demand and heater temperature was anticipated to effectively reduce the detection and concentrations of *L. pneumophila*. As the operating profiles changed sequentially from LD/LT to HD/LT and then from HD/LT to HD/HT, *L. pneumophila* had 17 days to acclimate to low water heater temperature compared to 10 days to acclimate to a higher temperature regardless of the water demand (Table 2). The lower acclimation period for a higher water heater setpoint might have affected the impact that a combined higher water demand and water heater temperature were expected to produce. During the LD/HT profile, *L. pneumophila* was detected in 5 % of the analyzed samples at concentrations ranging from 3.64 to 4.69 Log_10_ GC/100 mL (Table 2). The lognormal distribution provided a good fit to both censored and uncensored *L. pneumophila* measurements during the LD/HT profile (median = 0.79 Log_10_ GC/100 mL and 95th percentile = 3.34 Log_10_ GC/100 mL, Fig. 2 and Fig. S-10). A comparison of *L. pneumophila* concentrations and detection rates between the HD/HT and LD/HT profiles and the HD/LT and LD/HT profiles did not show significant differences (Table 5). However, the LD/HT profile had significantly lower concentrations and detections of *L. pneumophila* compared to the LD/LT profile (p-values = 0.09 and 0.06, respectively, Table 5). Consequently, increasing the water heater setpoint from 54 °C ± 3 °C to 66 °C ± 3 °C significantly reduced the concentrations and occurrence of *L. pneumophila* in the Net-Zero house plumbing system when the water demand was kept at a low rate. Gleason et al. (2023) found a significant association between hot water temperature (reported for the nearest location to hot water tanks) and *Legionella* spp. detection by culture methods when temperatures were categorized into low (<50.6 °C) and high (≥50.6 °C). However, Gleason et al. (2023) did not find a similar association between *Legionella* spp. detection by molecular methods (PCR) and hot water temperature.

Data from profiles LD/LT and HD/LT were combined into one profile referred to as LT. Similarly, data from profiles HD/HT and LD/HT were combined into one profile referred to as HT. While *L. pneumophila* was detected at a higher frequency as well as higher concentrations in profile LT compared to profile HT, these differences were not statistically significant (p-values = 0.253 and 0.236, respectively, Table 5). While merging the aforementioned profiles would be expected to reveal a stronger difference in *L. pneumophila* concentrations between low and high temperature settings, combining these profiles also entails merging high and low water demands in each of the resulting profiles (i.e., LD/LT & HD/LT and HD/HT & LD/HT). Having two different water demands (with competing effects) in each of the resulting profiles can mask the impact of temperature on the detection and concentration of *L. pneumophila*.

#### 3.2.2. P. aeruginosa

**Fig. 3.**
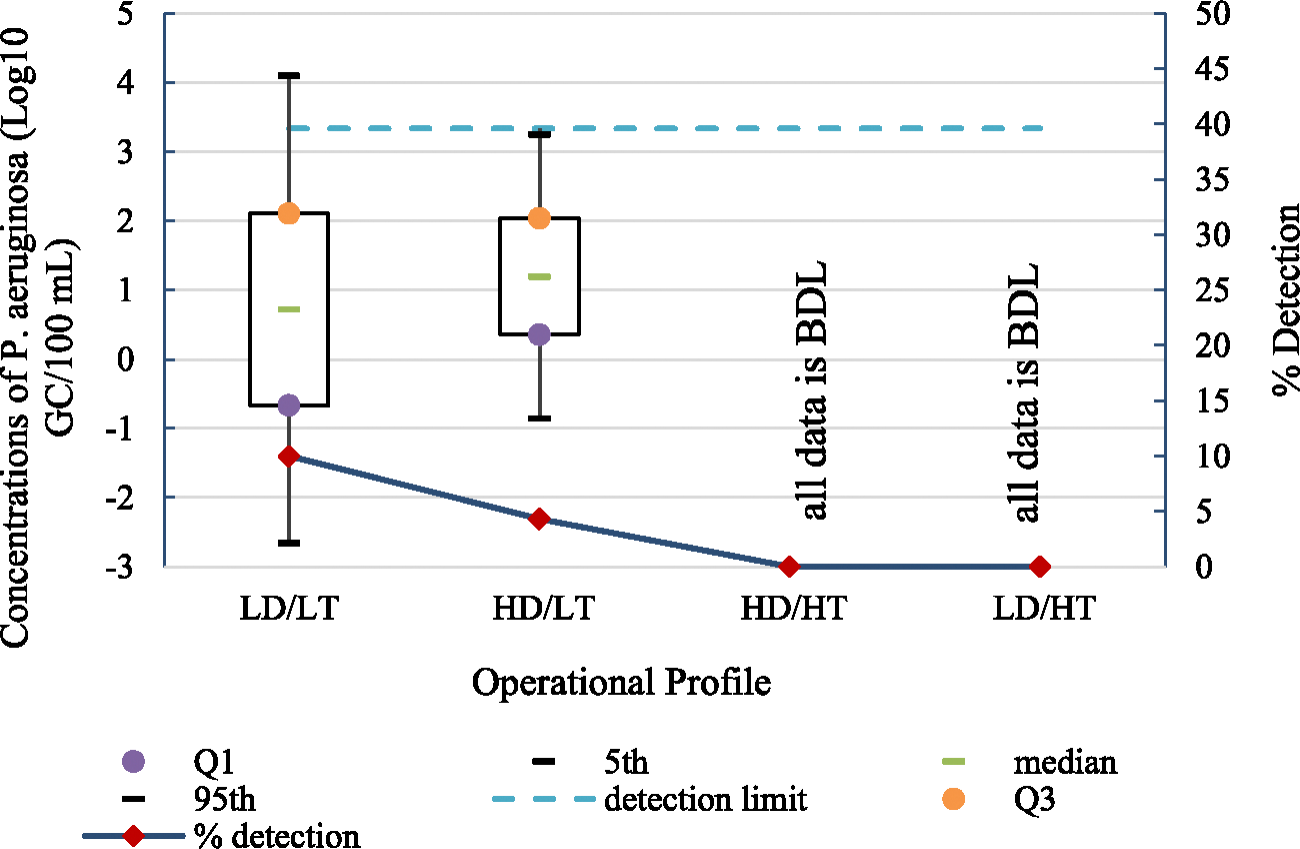
Boxplots of lognormal distribution Fit to *P. aeruginosa* concentrations during each operational profile (n_LD/LT_ = 60, n_HD/LT_ = 70, n_HD/HT_ = 70, and n_LD/HT_ = 60).

During the LD/LT profile, *P. aeruginosa* was detected in 10 % of the samples at concentrations ranging from 3.60 to 5.34 Log_10_ GC/100 mL (Table 2). A lognormal distribution was found to provide the best fit to *P. aeruginosa* data including both censored and uncensored observations (median = 0.72 Log_10_ GC/100 mL and 95th percentile = 4.10 Log_10_ GC/100 mL, Fig. 3 and Fig. S-11). For the HD/LT profile, *P. aeruginosa* occurred at a lower rate, i.e., 4.3 % and lower concentrations ranging from 3.48 to 4.16 Log_10_ GC/100 mL when compared to the LD/LT profile (Table 2). The lognormal distribution provided a good fit to both censored and uncensored measurements in the HD/LT profile (median = 1.20 Log_10_ GC/100 mL and 95th percentile = 3.25 Log_10_ GC/100 mL, Fig. 3 and Fig. S-12). Neither the concentrations nor the % detection of *P. aeruginosa* differed significantly between the LD/LT and HD/LT profiles (p-values = 0.18 and 0.30, respectively, Table 5).

*P. aeruginosa* was not detected in any of the samples collected during the HD/HT and LD/HT profiles (Table 2). Due to the high percentage of censored data, comparisons of *P. aeruginosa* concentrations between most profile pairings were not conducted (Table 5). Nonetheless, the occurrence of *P. aeruginosa* exhibited a significant decrease during the HD/HT and LD/HT profiles compared to the LD/LT profile (p-values = 0.01 and 0.03, respectively, Table 5). Therefore, increasing both the water demand and the water heater setpoint temperature resulted in significantly lower *P. aeruginosa* detections. Additionally, increasing the water heater setpoint temperature while maintaining a low water demand resulted in significantly lower *P. aeruginosa* detections. This demonstrates that *P. aeruginosa* and *L. pneumophila* had different responses to the change in operational conditions. Thus, an attempt to reduce the proliferation of one OPPP may not have the same impact on other OPPPs. No other significant differences were found in the % detection of *P. aeruginosa* for the remaining profile pairings.

Within the pooled profiles LT (i.e., LD/LT and HD/LT) and HT (i.e., HD/HT and LD/HT), the concentrations of *P. aeruginosa* could not be compared because the high number of non-detects in profile HT prevented the use of the MLE method. However, *P. aeruginosa* was detected in profile LT at a higher frequency compared to profile HT (p-value = 0.003, Table 5) further demonstrating the significant impact of water heater temperature on the occurrence of OPPPs.

#### 3.2.3. M. avium

*M. avium* was not detected in any of the samples collected during the LD/LT profile (Table 2). During both the HD/LT and HD/HT profiles, *M. avium* was found in 2.9 % of the samples at concentration ranges of 3.40 to 3.70 Log_10_ GC/100 mL and 3.60 to 3.99 Log_10_ GC/100 mL, respectively. During the LD/HT profile, *M. avium* was found in one sample from the kitchen sink at a concentration of 3.77 Log_10_ (GC/100 mL). Comparisons of *M. avium* concentrations between individual profile pairings were not possible due to the high percentage of censored data. Also, the % detection of *M. avium* did not differ significantly for any of the individual profile pairings.

Similar to *P. aeruginosa*, the concentrations of *M. avium* could not be compared between the pooled profiles LT and HT due to the high number of non-detects in profile LT. The occurrence of *M. avium* in profile LT did not differ significantly from that in profile HT (p-value = 1, Table 5). Ley et al. (2020) reported that lower water use led to higher stagnation, and the latter correlated with higher *Mycobacterium* spp. concentrations. The high percent of data below the analytical limit of detection in this study could have potentially masked any influence that the water demand and/or water heater setpoint might have on *M. avium*.

### 3.3. Characterization of OPPPs across different sampling sites and sample types (Cold vs. mixed)

#### 3.3.1. L. pneumophila

**Fig. 4.**
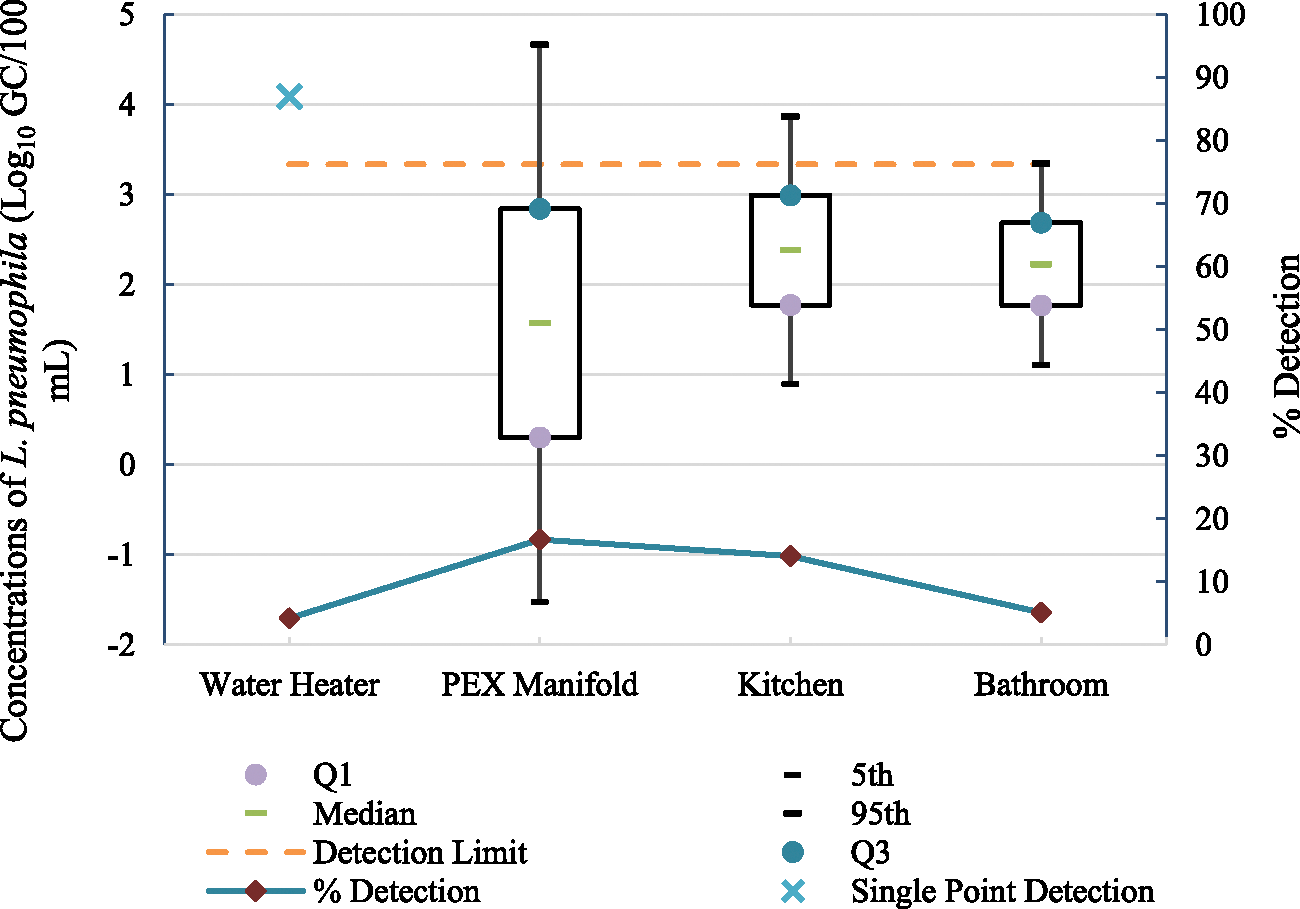
Boxplots of lognormal distribution fit to *L. pneumophila* concentrations and % detection throughout the entire study period at different sampling locations (n_water heater_ = 24, n_PEX manifold_ = 24, n_kitchen_ = 64, and n_bathroom_ = 136).

*L. pneumophila* was not detected in any of the samples collected from the influent water supply to the NZERTF. On the other hand, *L. pneumophila* was found in 4.2 %, 16.7 %, 14.1 %, and 5.1 % of the samples collected from the water heater, PEX manifold, kitchen, and bathroom, respectively, with the highest detections originating from the PEX manifold (Fig. 4). This could be because the PEX manifold includes a portion of a connecting pipe, which is considered a dead leg (i.e., section of pipe that is rarely used or unused). The presence of dead-legs has been identified as a factor contributing to the inoculation of premise plumbing by opportunistic pathogens including *Legionella* (Falkinham, 2022).

As water flowed from the PEX manifold in the basement to the kitchen on the first floor, no significant changes were observed in the concentration and % detection of *L. pneumophila* (p-values = 0.43 and 0.75, respectively, Table 5). However, as water flowed from the PEX manifold to the bathroom, *L. pneumophila* concentrations exhibited a significant increase (p-value = 0.02, Table 5) whereas the % detection exhibited a significant decrease (p-value = 0.06, Table 5). Additionally, samples collected from the first-floor kitchen had significantly higher concentrations and detections of *L. pneumophila* (p-values = 0.03 and 0.04, respectively) compared to the second-floor bathroom samples. This could indicate that the detection of *L. pneumophila* decreases as water flows away from the potential seeding source (PEX manifold) (Fig. 4). Pipe sections through which water flows from the PEX manifold to the kitchen and bathroom are less stagnant compared to the dead leg and therefore, exhibit lower *L. pneumophila* detection rates. Additionally, nutrients available to *L. pneumophila* at the seeding source might have been attenuated as water flowed away from the PEX manifold. It is important to note, however, that since molecular methods were applied for sample analysis, these detection rates reflect signals from both viable and non-viable *L. pneumophila*.

All *L. pneumophila* detections in bathroom samples originated from the faucet and tub with no detections in the samples collected from the shower. Codony et al. (2002) investigated the presence and concentration of *L. pneumophila* in the showers of 145 homes in Spain, 113 of which had patients with community-acquired Legionnaire’s disease. They found *L. pneumophila* in 6.9 % of the shower samples at a concentration range of 1.11 to 3.74 Log_10_ (GC/100 mL). Nonetheless, 3 of the 10 samples positive for *L. pneumophila* came from control homes with no patients. Hamilton et al. (2019) reported that showers are the highest drivers of *L. pneumophila* infection risk followed by faucets and toilets (showers > faucets > toilets). This demonstrates that the exposure to *L. pneumophila* in a residential setting can vary by site (i.e., faucet, tub, shower, etc.).

Within the context of the NZRTF, exposure sites are limited to the kitchen faucet, bathroom faucet, and bathtub since *L. pneumophila* was not detected in any of the water samples collected from the shower. Infection with *L. pneumophila* can occur through inhalation of contaminated aqueous aerosols produced by water fixtures (Hamilton et al., 2019). Bollin et al. (1985) evaluated contaminated hot water faucets for the aerosolization of *L. pneumophila* using a multistage cascade impaction air sampler. Their data showed that contaminated hot water faucets could produce fine particle aerosols containing *L. pneumophila* during routine use. The size of these aerosols is small enough to reach the lower respiratory system (Bollin et al., 1985).

**Fig. 5.**
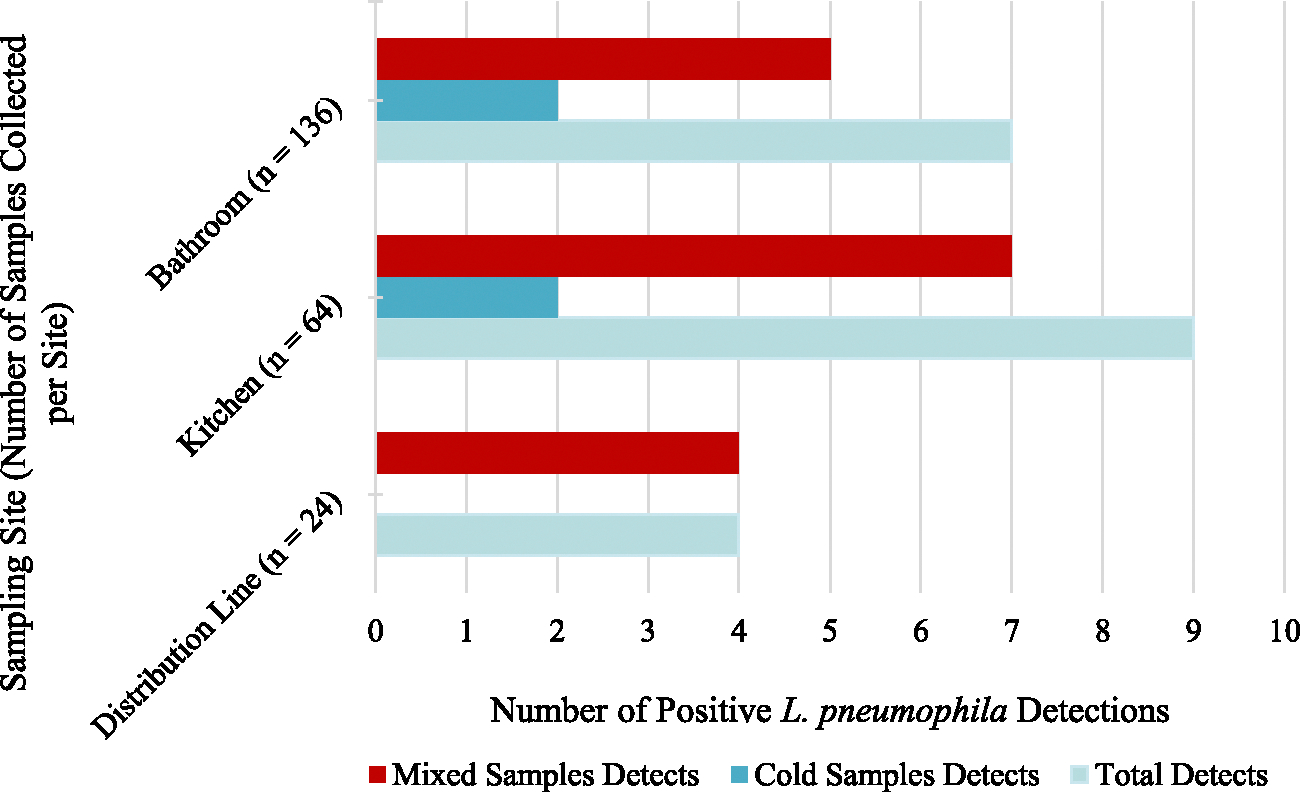
Distribution of *L. pneumophila* detections across sampling sites and sample types (Cold vs. Mixed).

The majority of *L. pneumophila* detections in bathroom and kitchen samples were found in mixed water samples (Fig. 5). *L. pneumophila* was found at significantly higher concentrations and detection rates in mixed water samples compared to cold ones (p-values = 0.03 and 0.02, respectively, Table 5). Studies investigating OPPPs in residential settings usually consider hot samples only (Gleason et al., 2023; Borella et al., 2004) or hot and cold samples (Ley et al., 2020); mixed samples are hardly ever considered. The definition of hot versus mixed samples can differ based on the study setting and conditions. Thus, comparing detections and concentrations among studies based on sample type (i.e., cold, mixed, and hot) might be misleading.

#### 3.3.2. P. aeruginosa and M. avium

**Fig. 6.**
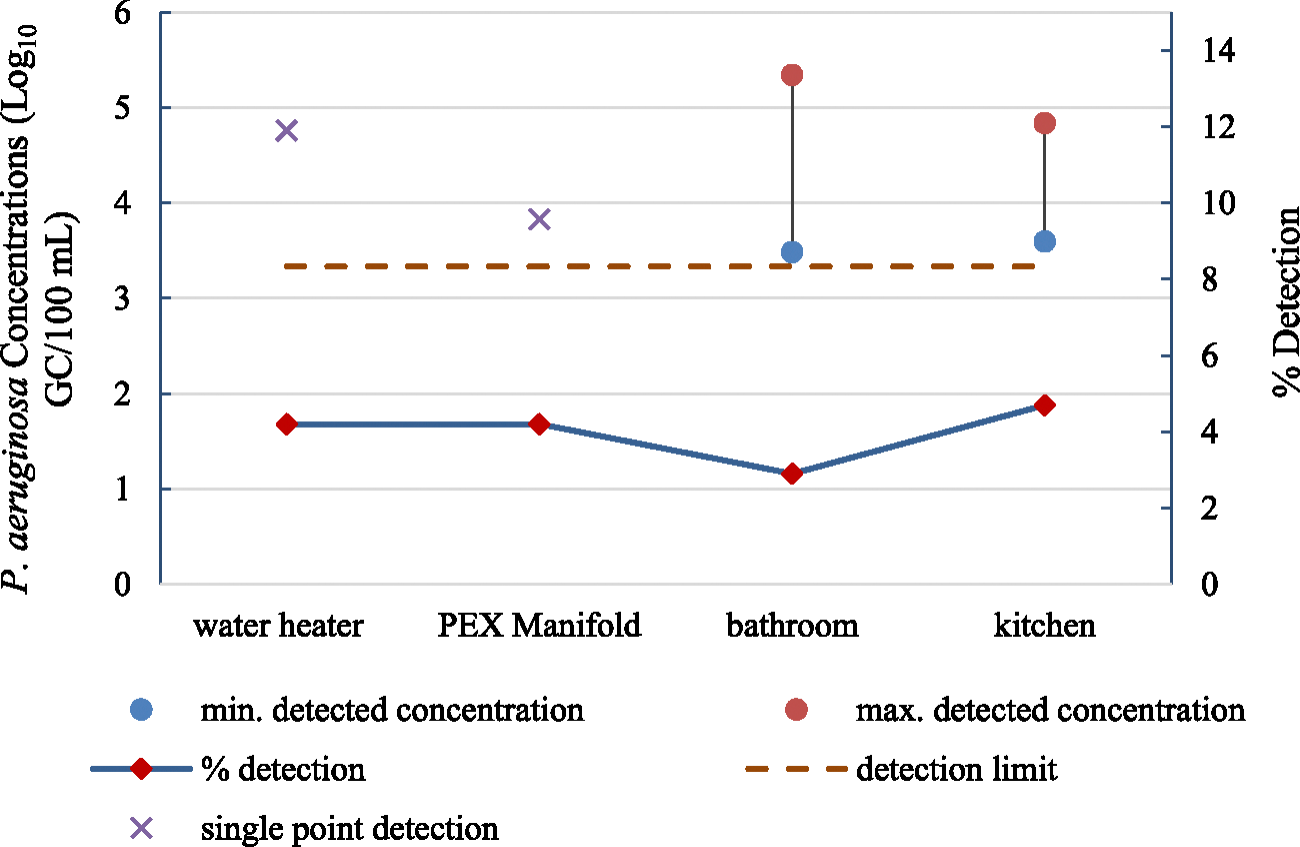
Percent detection and concentration range for *P. aeruginosa* throughout the entire study period at different sampling locations (n_water heater_ = 24, n_PEX manifold_ = 24, n_kitchen_ = 64, and n_bathroom_ = 136).

*P. aeruginosa* was not detected in any of the samples collected from the influent water supply to the NZERTF. While *P. aeruginosa* was detected at a higher percentage in kitchen samples (4.7 %) compared to bathroom (2.9 %), heat-pump water heater (4.2%), and PEX manifold samples (4.2 %), these differences were not statistically significant (Fig. 6 and Table 5). As mentioned earlier, Wang et al. (2012) reported the occurrence of *P. aeruginosa* in 1.1 % of the samples they investigated. This detection originated from a water heater. However, the concentration of *P. aeruginosa* detected in the water heater in this study (1 out of 24 samples, Fig. 6) is about two and a half orders of magnitude higher than that reported by Wang et al. (2012).

The high percentage of censored data prevented the use of the MLE method to compare *P. aeruginosa* concentrations between pairings of different sampling sites. No significant differences were found in the concentrations and % detection of *P. aeruginosa* between mixed and cold samples (p-values 0.58 and 1, respectively, Table 5). Similar to *L. pneumophila* and *P. aeruginosa, M. avium* was not found in any of the samples collected from the influent water supply. *M. avium* detection did not vary significantly between different pairings of sampling sites and sample types (Table 5).

### 3.4. Potential confounding variables

This study focused on investigating the effect of water demand, water heater temperature, sampling sites, and sample type on the occurrence and concentrations of OPPPs. However, factors such as the incoming water source, pipe material, and maintenance practices can potentially influence the growth and proliferation of these pathogens. The investigated OPPPs were not found in any of the samples collected from the influent water pipe. As a result, the water source and distribution network conveying water to the NZERTF were not contributing factors to the occurrence of OPPPs under the conditions of this study. Rhoads et al. (2020) collected water samples from different locations within the premise plumbing of 27 households in Flint, MI including samples representative of influent water entering households from the distribution system. Twenty households had quantifiable concentrations of *Legionella* spp. in the samples representative of influent water. These households exhibited significantly more *L. pneumophila* and *Legionella* spp. genetic material within premise plumbing compared to households without quantifiable *Legionella* spp. in influent water (Rhoads et al., 2020). Thus, while source water and/or the distribution network can impact the proliferation of OPPPs in indoor residential plumbing, OPPPs can still be detected in premise plumbing despite their absence in municipal water.

Pipes at the NZERTF are made of cross-linked polyethylene (PEX). This material can release organic carbon used for microorganisms’ growth (Cullom et al., 2020), which might have contributed to the occurrence of OPPPs within the NZERTF’s plumbing. On the other hand, plastic pipe material has lower chlorine demand (Cullom et al., 2020). This further demonstrates the complexity involved in the interactions among the different factors and mechanisms impacting the growth of OPPPs. Common maintenance practices to reduce/eliminate OPPPs include flushing and water heater cleaning. Specifically, flushing premise plumbing pipes can be used as both a corrective measure and a preventative maintenance tool to limit the occurrence of OPPPs in indoor plumbing (Water Research Foundation (WRF), 2024). Rhoads et al. (2020) demonstrated that a one-time cleanout of accumulated sediments in residential water heaters in Flint, MI had temporarily reduced the detection and concentrations of *L. pneumophila* and *Legionella* spp. Premise plumbing at the NZERTF, which was built in 2012, has never been flushed. The electric heater at the NZERTF was replaced two years before the study commenced, and the replacement heater has never been cleaned. The lack of maintenance practices at the NZERTF could potentially have contributed to OPPPs growth. Therefore, homeowners should be alerted to the importance of flushing indoor plumbing and cleaning their water heaters.

### 3.5. Suggested measures to address the presence of OPPPs in indoor premise plumbing

Warm environments can promote the growth of OPPPs including *L. pneumophila* (Furst et al., 2024). For instance, the ideal temperature for the proliferation of *L. pneumophila* ranges from 25 to 45 °C (Katz & Hammel, 1987). Thus, raising the water heater setpoint temperature is a potential measure to reduce the levels of OPPPs in residential premise plumbing (Falkinham et al., 2015). The CDC recommends the storage of hot water at a temperature above 60 °C (Centers for Disease Control (CDC), 2025). In this study, *L. pneumophila* was detected at significantly lower frequencies and concentrations when the water heater setpoint was raised from 54 °C to 66 °C even when the water demand remained low. The occurrence of *P. aeruginosa* decreased significantly when the water heater temperature set point was raised from 54 °C to 66 °C and the water demand was increased. The occurrence of *P. aeruginosa* followed a similar trend when the water heater temperature setpoint was raised while the demand was kept low. This demonstrates that increasing the water heater setpoint temperature can significantly reduce the detection and concentration of OPPPs.

Reduced water demand results in an increased water age and stagnation within premise plumbing (Rhoads et al., 2016). Higher water age is associated with the loss of disinfectant residual (Rhoads et al., 2016) which leads to higher proliferation of OPPPs. In this study, increasing the water demand while fixing the water heater at the lower temperature setting resulted in a significant decrease in *L. pneumophila* detection and concentrations. To counter the negative impact that water conservation measures might have on the growth of OPPPs, point-of-use water treatment units such filters (Falkinham et al., 2015) or UV disinfection can be installed at fixtures to reduce/eliminate OPPPs. However, filter cartridges and UV lamps should be replaced at the intervals recommended by the manufacturer to ensure proper performance. Flushing premise plumbing can help reduce biofilms in pipes, re-optimize the water temperature, and replenish disinfectant residuals (Water Research Foundation (WRF), 2024). Additionally, routine flushing and water heater cleaning can help limit the growth of OPPPs.

## 4. Limitations and conclusions

Despite the study’s goal to investigate the influence of water demands and water heater setpoint temperature on the detection and concentration of OPPPs, the final dataset remains limited due to the low detection of OPPPs. This prevents the definitive identification of strong relationships between OPPPs and the investigated operational profiles, sampling sites, and sample types. Nonetheless, some significant trends were observed for *L. pneumophila* and *P. aeruginosa*. Low detection frequencies of OPPPs in residential plumbing is a challenge that will likely persist due to pragmatic considerations related to the sampling volume, the absence of OPPPs in most feed waters, and water heaters’ tank hydraulics. Data collected in the literature to date suggest that OPPPs occur at a low frequency within residential premise plumbing, but that concentrations are notable when OPPPs are detected. Further limitations specific to this study include the high water heater setpoint temperatures and the age of the residential test facility (less than ten years).

The absence of OPPPs in the water supply to the NZERTF did not negate the subsequent growth and proliferation of *L. pneumophila, P. aeruginosa*, and *M. avium* in the heat-pump water heater, PEX manifold, and/or distal taps. Faucets posed a higher risk of exposure to *L. pneumophila* compared to tubs and showers within the NZERTF. Water demand and the water heater setpoint temperature were found to be independently associated with the occurrence and concentrations of *L. pneumophila* in the NZERTF’s plumbing system. Water demand and water heater setpoint temperature were both associated with the occurrence of *P. aeruginosa* in the plumbing system of the NZERTF. The detection frequency of *L. pneumophila* decreased significantly as water flowed from a potential seeding/growth source (PEX manifold) to the farthest sampling location (bathroom) within the plumbing system.

## Supplementary Material

Supplementary Material

## Figures and Tables

**Table 1:** Description of operational profiles and duration of acclimation and sampling periods (2021 Dates).

Profile abbreviation	Profile description	Acclimation Period	Sampling dates

LD/LT	Low water demand/Low water temperature	9/20 to 9/26	9/27, 9/29, 10/4 and 10/6
HD/LT	High water demand/Low water temperature	10/9 to 10/18	10/19 and 10/26
HD/HT	High water demand/High water temperature	10/30 to 11/8	11/9 and 11/16
LD/HT	Low water demand/High water temperature	11/20 to 11/28	11/29, 12/1, 12/6 and 12/8

**Table 2:** Percent detection and concentration range of OPPPs (Log_10_ GC/100 mL) in water samples collected during the study^a^.

Operational Period (2021 dates)	Acclimation Period (days)	No. of Collected Samples	*L. pneumophila*	*P. aeruginosa*	*M. avium*

9/27 – 10/6 (LD/LT)	7	60	15 % (3.60 – 4.98)	10 % (3.60 – 5.34)	0 %
10/19 – 10/26 (HD/LT)	10	70	5.7 % (3.40 – 3.95)	4.3 % (3.48 – 4.16)	2.9 % (3.40 – 3.70)
11/9 – 11/16 (HD/HT)	10	70	7.1 % (3.40 – 4.17)	0 %	2.9 % (3.60 – 3.99)
11/29 – 12/8 (LD/HT)	9	60	5 % (3.64 – 4.69)	0 %	1.7 % (3.77^c^)
9/27 – 12/8 (entire study period)	NA^b^	260	8.1 % (3.40 – 4.98)	3.5 % (3.48 – 5.34)	1.9 % (3.40 – 3.99)

a the concentration range is for positive samples above a detection limit of 3.33 Log_10_ (GC/100 mL) for all OPPPs

b: an acclimation period was applied for each operational profile separately and is thus not applicable for the entire study period

c: single sample detection.

**Table 3:** Percent detection and concentration of OPPPs in national and international studies.

Study / No. of samples	Location	Setting	Sample type	Sampled sites	*L. pneumophila* % Detected (Ranges of concentration)		*Legionella* spp. % Detected (Ranges of concentration)	
		
					Molecular^a^	Culture^b^	Molecular^a^	Culture^b^

this study / n = 260	Maryland, USA	NZERTF test facility^c^	cold, hot, and mixed	influent, heater, PEX manifold, kitchen and bathroom faucets, tub, and shower	8.1 % (3.40 – 4.98)	NA^d^	NA^d^	NA^d^
Ley et al. (2020)/n = 259	Indiana, USA	ReNEWW house^e^	hot and cold	service line; cold water lines: kitchen sink and distal end bathroom sink; hot water lines: heater, kitchen sink, distal end bathroom sink, and distal end shower	0%	NA^d^	78 % (1.03 – 5.72)	NA^d^
Gleason et al. (2023) / n = 451	New Jersey, USA	94 residential households^f^	hot	showers, hot tubs, and infrequently used taps	NR^g^	4.4 % (0.7 – 3.8)^h^	37.9 %^h^ (NR^g^)	7.3 %^h^ (NR^g^)
Wang et al. (2012)/n = 90	Virginia, USA	29 residential households^i^	NR^g^	taps and water heaters	4.4 % (2.99^j^)	NA^d^	30 % (4.27^j^)	NA^d^
Borella et al. (2004)/n = 146	Bari, Bologna, Milan, Modena, Naples, and Rome; Italy	private homes (apartments, houses, and villas)	hot	shower heads and bathroom taps	NA^d^	17.1% (1.1 – 3.54)^k, l^	NA^d^	22.6 % (0.73 – 3.61)^k^
Codony et al. (2002)/n = 145	Catalon territory, Spain	145 homes^m^	NR^g^	showers	NA^d^	6.9 %^n^(1.11 – 3.74)	NA^d^	10.3 %

a: units are in Log_10_ (GC/100 mL)

b: units are in Log_10_ (CFU/100 mL)

c: Net-Zero Energy Residential Test Facility

d: not applicable

e: Retrofitted Net-Zero Energy, Water, and Waste house

f: 37 homes had Legionnaire’s disease case patients

g: not reported

h: excluding outside tap and hot tub samples

i: the selected households represent a range of water travel time from the drinking water treatment plant to households

j: average of positive samples

k: the range represents the 5th and 95th percentiles

l: Borella et al. (2004) reported the percentiles for *L. pneumophila* serogroup 1 and *L. pneumophila* serogroups 2 – 14. The range reported herein was obtained by summing the values reported by Borella et al. (2004) for *L. pneumophila* serogroup 1 and *L. pneumophila* serogroups 2 – 14

m: 113 homes had patients with community-acquired Legionnaire’s disease

n: 7 of the 10 samples positive for *L. pneumophila* came from case homes.

**Table 4:** Concentrations of *L. pneumophila* in kitchen and bathroom faucet samples in contrast with infection critical concentrations at different risk benchmarks.

Risk Target Value for Conventional Faucets	Infection Critical Concentrations (Log_10_ CFU/100 mL) Calculated by Hamilton et al. (2019)			NZERTF Faucet Concentrations (Log_10_ GC/100 mL)		
		
	5th percentile	median	95th percentile	5th percentile	median	95th percentile

10^−4^ annual^a^	−1.26	1.08	3.59	0.80	2.21	3.63
10^−4^ per exposure^b^	2.61	4.94	7.45			
10^−6^ DALY annual^c^	−3.25	−0.91	1.60			
10^−6^ DALY per exposure^d^	0.62	2.96	5.46			

**Table 5:** Assessment of significant differences in OPPPs concentrations and % detection between different group pairings (Bold-faced p-values indicate a significant difference at the 90 % confidence level).

Category	Group Pairings	p-values for *L. pneumophila*		p-values for *P. aeruginosa*		p-values for *M. avium*	
			
		Concentration^a^	% Detection^b^	Concentration^a^	% Detection^b^	Concentration^a^	% Detection^b^

Operational Conditions	Profiles LD/LT and HD/LT	**0.072**	**0.077**	0.176	0.301	NA^c^	0.499
	Profiles LD/LT and HD/HT	0.118	0.149	NA^c^	**0.008**		0.499
	Profiles LD/LT and LD/HT	**0.093**	**0.062**		**0.027**		1
	Profiles HD/LT and HD/HT	0.727	1		0.245		1
	Profiles HD/LT and LD/HT	0.958	1		0.249		1
	Profiles HD/HT and LD/HT	0.712	0.725		NA^d^		1
	Profiles LT and HT^e^	0.236	0.253	NA^c^	0.003	NA^c^	1
Sampling Site	Water Heater and PEX manifold	NA^c^	0.348	NA^c^	NA^d^	NA^c^	NA^d^
	Water Heater and Kitchen		0.274		1		1
	Water Heater and Bathroom		1		0.561		1
	PEX manifold and Kitchen	0.426	0.745		1		1
	PEX manifold and Bathroom	**0.022**	**0.063**		0.561		1
	Kitchen and Bathroom	**0.029**	**0.037**		0.682		0.656
Sample Type	mixed and cold	**0.025**	**0.018**	0.582	1	NA^c^	0.387
	mixed and hot	NA^c^	0.474	NA^c^	1	NA^c^	1
	hot and cold		1		0.569		1

a The MLE method was used to determine whether the concentrations of each OPPP were significantly different between group pairings

b: The chi-square test was used to determine whether the detection (occurrence) of each OPPP was significantly different between group pairings unless the expected tabular cell count in contingency tables was less than 5. For expected counts < 5, Fisher’s exact test was used

c: Comparing the concentrations between different group pairings was not conducted because the high percentage of data below the detection limit prohibited the use of the MLE method

d: Fisher’s Exact Test could not be conducted because data was identical in both groups; Profile LT is a combination of profiles LD/LT and HD/LT, and profile HT is a combination of profiles HD/HT and LD/HT.

## Data Availability

Data will be made available on request.

## Synopsis:

Minimal research exists on opportunistic pathogens in residential settings. This study may help the plumbing industry improve practices for building water systems to control the growth of OPPPs.
