# Supplementary material for: Impacts of household water demands and water heater delivery temperatures on opportunistic premise plumbing pathogens (OPPPs) in a residential setting
Source: Total Environ Microbiol. Author manuscript; Available in PMC 2026 May 14. (PMC13170537; doi:10.1016/j.temicr.2025.100002)
Supplement: Supplementary Material [file NIHMS2064954-supplement-Supplementary_Material.docx]

**Supporting Information Section**

**Treatment at the Potomac and Patuxent Water Filtration Plants:**

The Potomac Water Filtration Plant (WFP) draws water from the Potomac River and produces ~120 million gallons per day, while the Patuxent WFP draws water from the Duckett Reservoir and produces 60 million gallons per day. The water treatment process is a full scale one, and the treatment train includes: coagulation/flocculation, sedimentation, filtration, granular media filters, primary disinfection (utilizes ultraviolet light radiation) and secondary residual disinfection (utilizes free chlorine), then corrosion control chemicals (orthophosphate and lime) are added to the finished water and sent to the water storage tank for high demand periods. Secondary residual disinfection is utilized to maintain a residual throughout the distribution system to reduce bacterial re-growth as well as to control biofilm formation. Chlorine can be broken down into three parts: free, combined, and total. Free chlorine is available for controlling microbial regrowth, survival, and monitoring purposes; while combined chlorine is the chlorine that binds to any contaminants in the water; and total chlorine is the sum of free and combined chlorine.

**Operation and Temperature Measurements of the Heat Pump Water Heater at the NZERTF**

The water heater was in operation for two years before the experiments in this study. During the heating cycles, the heat pump was designed to draw water from the bottom of the tank and heated water was returned to the top. These temperatures were chosen for two reasons: a) the heat-pump water heater lowest temperature setting was 54 °C; b), the heat-pump water heater was set to 66 °C to reduce some OPPPs growth even though this ‘higher’ temperature setting is not typically used in residential buildings. In addition, internal tank temperature measurement was not possible during the sampling period. Therefore, thermocouples located at the water heater outlet were used to monitor delivery temperatures with an uncertainty at the 95 % confidence level of ± 0.2 °C (0.4 °F). Hot water exiting the water heater entered a thermostatic mixing valve set to 49 °C (120 °F) to prevent scalding of building occupants. Cold and hot water were delivered to the fixtures and water-utilizing appliances via two manifolds and 1 cm (0.38 in) nominal diameter cross-linked polyethylene (PEX) tubing (Figure S-1). The hot water PEX tubing was covered in 1.9 cm (0.75 in) polyethylene foam insulation (R-value 0.53 K·m^2^/W (3 h·ft^2^·°F/Btu)) to mitigate heat loss. The lengths of the hot and cold PEX tubing, as well as the average mixed water flow rates, to each fixture tested are provided Table S-1. The ratio of hot to cold water per use varied as each fixture was manually adjusted during the set-up of each experiment to deliver water at a specific average temperature: approximately 41 °C (105 °F) at the sinks and 43 °C (110 °F) at the showers and bathtubs (ASHRAE, 2019).

**The Maximum Likelihood Estimation Method**

The MLE method was employed by coding the concentrations of OPPPs to account for censoring at the limit of detection (LOD) as (0, LOD) for censored data. For uncensored data, concentrations higher than the limit of detection were coded using an interval with the same start and end, e.g., (2500, 2500) for a concentration value of 2500 GC/100 mL. The MLE method relies on observed uncensored concentrations and the proportions of data below the detection limit (Helsel, 2012). Criteria used to select the best distributional fit to concentrations of OPPPs within different data grouping were as follows:

- The Anderson-Darling (AD) value; the lower the AD value, the better.

Visual inspection of the fit to concentration data.


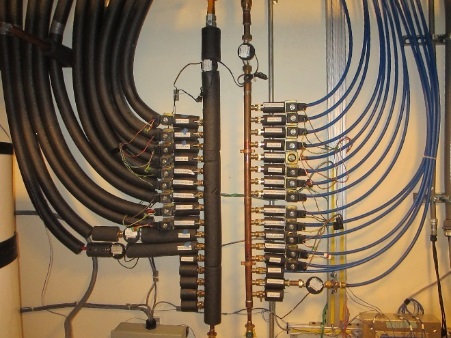


**Figure S-1. Hot manifold (left) and cold manifold (right), and PEX tubing leading to first and second floor fixtures**


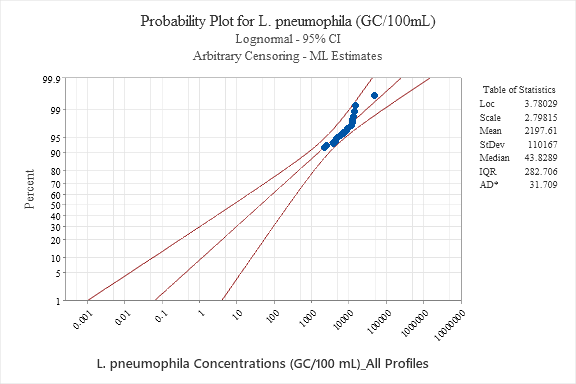


**Figure S-2. Lognormal Probability Plot for L. pneumophila Concentrations in Water Samples Collected across the Entire Study Period (The Upper and Lower Red Lines Represent the 95% Confidence Interval, n = 260 Data Points)**


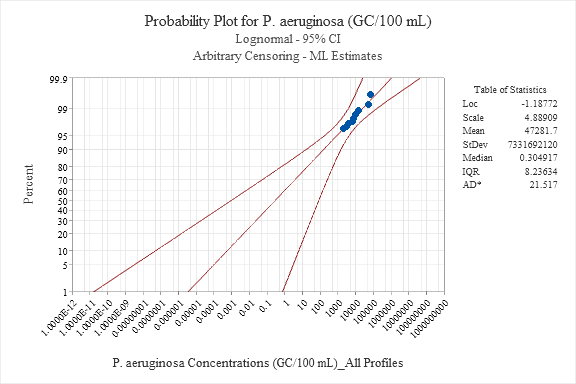


**Figure S-3. Lognormal Probability Plot for P. aeruginosa Concentrations in Water Samples Collected across the Entire Study Period (The Upper and Lower Red Lines Represent the 95% Confidence Interval, n = 260 Data Points)**


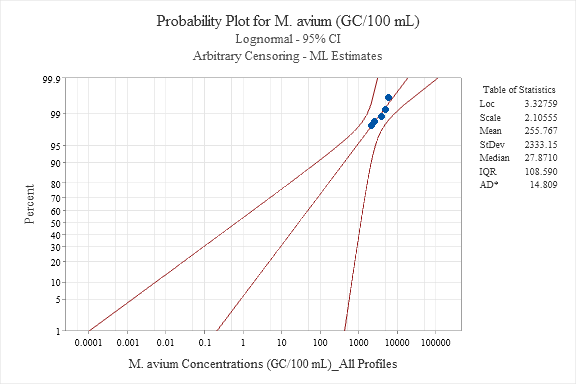


**Figure S-4. Lognormal Probability Plot for M. avium Concentrations in Water Samples Collected across the Entire Study Period (The Upper and Lower Red Lines Represent the 95% Confidence Interval, n = 260 Data Points)**

**Figure S-5. Boxplots of Lognormal Distribution Fit to the Concentrations of OPPPs and % Detection throughout the Entire Study Period (number of samples for each OPPP = 260)**


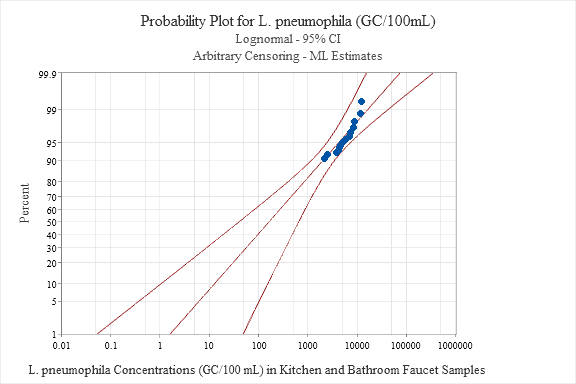


**Figure S-6. Lognormal Probability Plot for L. pneumophila Concentrations (GC/100 mL) in Water Samples Collected from Kitchen and Bathroom Faucets across the Study Period (The Upper and Lower Red Lines Represent the 95% Confidence Interval, n = 160 Data Points)**


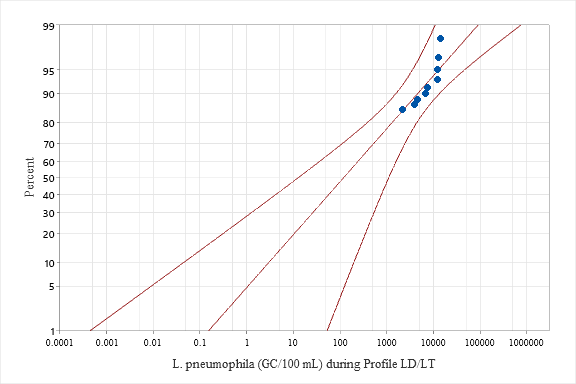


**Figure S-7. Lognormal Probability Plot for L. pneumophila Concentrations (GC/100 mL) in Water Samples Collected during the LD/LT profile (The Upper and Lower Red Lines Represent the 95% Confidence Interval, n = 60 Data Points)**


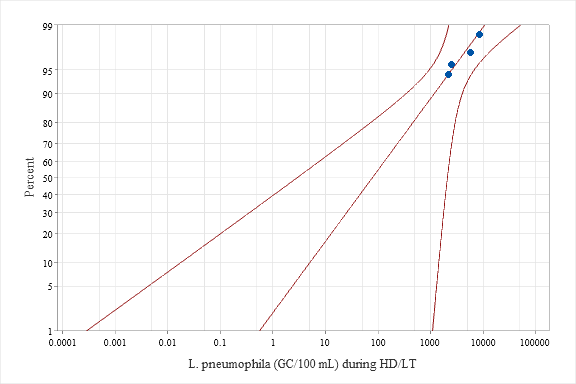


**Figure S-8. Lognormal Probability Plot for L. pneumophila Concentrations (GC/100 mL) in Water Samples Collected during the HD/LT profile (The Upper and Lower Red Lines Represent the 95% Confidence Interval, n = 70 Data Points)**


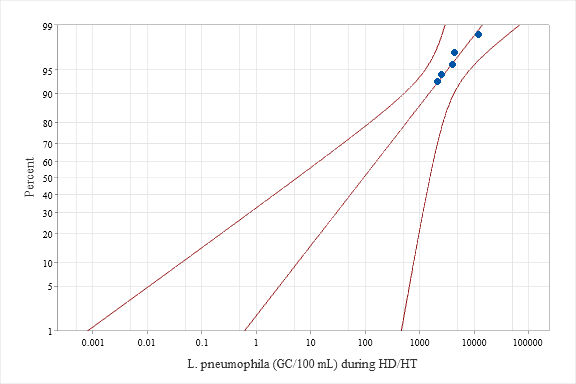


**Figure S-9. Lognormal Probability Plot for L. pneumophila Concentrations (GC/100 mL) in Water Samples Collected during HD/HT profile (The Upper and Lower Red Lines Represent the 95% Confidence Interval, n = 70 Data Points)**


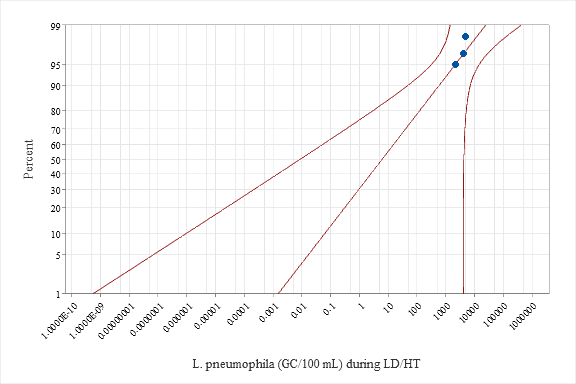


**Figure S-10. Lognormal Probability Plot for L. pneumophila Concentrations (GC/100 mL) in Water Samples Collected during the LD/HT profile (The Upper and Lower Red Lines Represent the 95% Confidence Interval, n = 60 Data Points)**


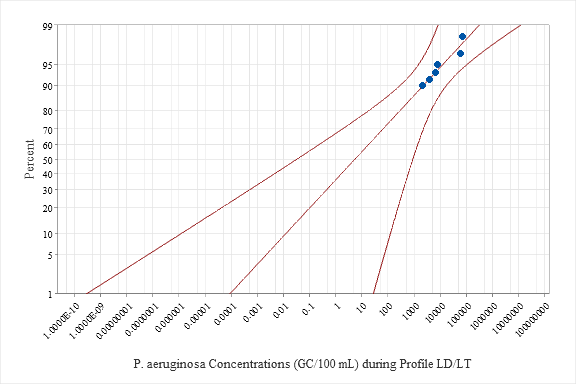


**Figure S-11. Lognormal Probability Plot for P. aeruginosa Concentrations (GC/100 mL) in Water Samples Collected during the LD/LT profile (The Upper and Lower Red Lines Represent the 95% Confidence Interval, n = 60 Data Points)**


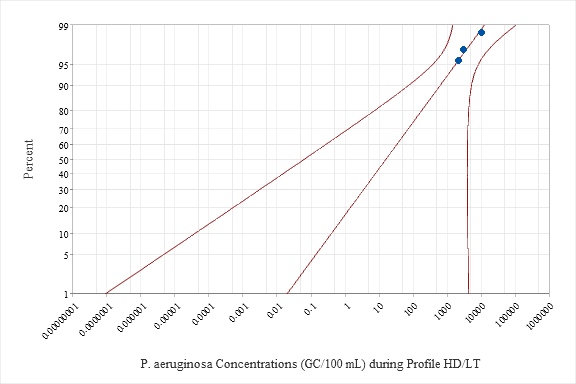


**Figure S-12. Lognormal Probability Plot for P. aeruginosa Concentrations (GC/100 mL) in Water Samples Collected during the HD/LT profile (The Upper and Lower Red Lines Represent the 95% Confidence Interval, n = 70 Data Points)**

**Table S-1. Details for Fixtures Utilized in Occupant Simulation for This Study^a^**

| Fixture | Hot PEX length  m (ft) | Cold PEX length  m (ft) | Average Mixed Water Flow Rate  Lpm, liters per min (gpm, gallons per min) | Target Mixed Water Volume per Draw  L (gal) |
| --- | --- | --- | --- | --- |
| Kitchen Sink | 8.33 (27.3) | 8.04 (26.4) | 3.8 (1.0) | 2.0 (0.54) |
| Owner’s Bath Sink | 10.2 (33.4) | 10.6 (34.6) | 6.1 (1.6) | 2.0 (0.54) |
| Owner’s Bath Shower | 12.8 (41.9) | 13.4 (44.1) | 7.6 (2.0) | 32.4 (8.57) |
| Owner’s Bathtub | 11.3 (36.9) | 11.7 (38.5) | 12 (3.3) | 112.8 (29.79) |

a: There are eight point-of-use locations in the house, but only four were sampled since the other four were not regularly used.

**Table S-2. Water Use Schedule for the Simulated Low Water Demand.**

| **Time** | **Monday** | **Tuesday** | **Wednesday** | **Thursday** | **Friday** | **Saturday** | **Sunday** |
| --- | --- | --- | --- | --- | --- | --- | --- |
|  |  |  |  |  |  |  |  |
| Baseline before use | **Samples:**  Influent  Cold water distribution  Top of water heater  Bottom of water heater  Mixed water manifold | N/A | **Samples:**  Influent  Cold water distribution  Top of water heater  Bottom of water heater  Mixed water manifold | N/A | | N/A | |
| 8:00 AM | **Sample:** Control (first flush) | Shower use (mixed) | **Sample:** Control (first flush) | Shower use (mixed) | | No usage | |
|  | Shower use (mixed) |  | Tub use (mixed) |  |  |  |  |
| ~8:10 AM | **Sample:** Experiment (mixed) After usage |  | **Sample:** Experiment (mixed) After usage |  |  |  |  |
| 8:20 AM | **Sample:** Control (first flush) | Bathroom faucet use (cold) | **Sample:** Control (first flush) | Bathroom faucet use (cold) | |  |  |
|  | Bathroom faucet use (cold) |  | Bathroom faucet use (cold) |  |  |  |  |
| ~8:25 AM | **Sample:** Experiment (cold) After usage |  | **Sample:** Experiment (cold) After usage |  |  |  |  |
| 8:30 AM | **Sample:** Control (first flush) | Bathroom faucet use (mixed) | **Sample:** Control (first flush) | Bathroom faucet use (mixed) | |  |  |
|  | Bathroom faucet use (mixed) |  | Bathroom faucet use (mixed) |  |  |  |  |
| ~8:31 AM | **Sample:** Experiment (mixed) After usage |  | **Sample:** Experiment (mixed) After usage |  |  |  |  |
| 8:40 AM | **Sample:** Control (first flush) | Kitchen faucet use (cold) | **Sample:** Control (first flush) | Kitchen faucet use (cold) | |  |  |
|  | Kitchen faucet use (cold) |  | Kitchen faucet use (cold) |  |  |  |  |
| ~8:45 AM | **Sample:** Experiment (cold) After usage |  | **Sample:** Experiment (cold) After usage |  |  |  |  |
| 8:50 AM | **Sample:** Control (first flush) | Kitchen faucet use (mixed) | **Sample:** Control (first flush) | Kitchen faucet use (mixed) | |  |  |
|  | Kitchen faucet use (mixed) |  | Kitchen faucet use (mixed) |  |  |  |  |
| ~8:51 AM | **Sample:** Experiment (mixed) After usage |  | **Sample:** Experiment (mixed) After usage |  |  |  |  |
| 6:40 PM | Kitchen faucet use (cold) | Kitchen faucet use (cold) | Kitchen faucet use (cold) | Kitchen faucet use (cold) | |  |  |
|  |  |  |  |  |  |  |  |
| 6:50 PM | Kitchen faucet use (mixed) | Kitchen faucet use (mixed) | Kitchen faucet use (mixed) | Kitchen faucet use (mixed) | |  |  |
|  |  |  |  |  |  |  |  |
| 9:20 PM | Bathroom faucet use (cold) | Bathroom faucet use (cold) | Bathroom faucet use (cold) | Bathroom faucet use (cold) | |  |  |
|  |  |  |  |  |  |  |  |
| 9:30 PM | Bathroom faucet use (mixed) | Bathroom faucet use (mixed) | Bathroom faucet use (mixed) | Bathroom faucet use (mixed) | |  |  |
|  |  |  |  |  |  |  |  |

**Table S-3. Water use schedule for the simulated high water demand.**

| **Time** | **Monday** | **Tuesday** | **Wednesday** | **Thursday** | **Friday** | **Saturday** | **Sunday** |
| --- | --- | --- | --- | --- | --- | --- | --- |
|  |  |  |  |  |  |  |  |
| Baseline before use | N/A | **Samples:**  Influent  Cold water distribution  Top of water heater  Bottom of water heater  Mixed water manifold | N/A | | | | |
| 8:00 AM | Shower use (mixed) | **Sample:** Control (first flush) | Shower use (mixed) | | | | |
|  |  | Shower use (mixed) |  |  |  |  |  |
| ~8:10 AM |  | **Sample:** Experiment (mixed) After usage |  |  |  |  |  |
|  |  |  |  |  |  |  |  |
| 8:20 AM | Shower use (mixed) | **Sample:** Control (first flush) | Shower use (mixed) | | | | |
|  |  | Shower use (mixed) |  |  |  |  |  |
| ~8:30 AM |  | **Sample:** Experiment (mixed) After usage |  |  |  |  |  |
|  |  |  |  |  |  |  |  |
| 8:40 AM | Tub use (mixed) | **Sample:** Control (first flush) | Tub use (mixed) | | | | |
|  |  | Tub use (mixed) |  |  |  |  |  |
| ~8:50 AM |  | **Sample:** Experiment (mixed) After usage |  |  |  |  |  |
|  |  |  |  |  |  |  |  |
| 9:10 AM | Bathroom faucet use (cold) | **Sample:** Control (first flush) | Bathroom faucet use (cold) | | | | |
|  |  | Bathroom faucet use (cold) |  |  |  |  |  |
| ~9:15 AM |  | **Sample:** Experiment (cold) After usage |  |  |  |  |  |
|  |  |  |  |  |  |  |  |
| 9:20 AM | Bathroom faucet use (mixed) | **Sample:** Control (first flush) | Bathroom faucet use (mixed) | | | | |
|  |  | Bathroom faucet use (mixed) |  |  |  |  |  |
| ~9:21 AM |  | **Sample:** Experiment (mixed) After usage |  |  |  |  |  |
|  |  |  |  |  |  |  |  |
| 9:30 AM | Bathroom faucet use (cold) | Bathroom faucet use (cold) | Bathroom faucet use (cold) | | | | |
|  |  |  |  |  |  |  |  |
| 9:40 AM | Bathroom faucet use (mixed) | Bathroom faucet use (mixed) | Bathroom faucet use (mixed) | | | | |
|  |  |  |  |  |  |  |  |
| 9:50 AM | Bathroom faucet use (cold) | Bathroom faucet use (cold) | Bathroom faucet use (cold) | | | | |
|  |  |  |  |  |  |  |  |
| 10:00 AM | Bathroom faucet use (mixed) | Bathroom faucet use (mixed) | Bathroom faucet use (mixed) | | | | |
|  |  |  |  |  |  |  |  |
| 10:10 AM | Bathroom faucet use (cold) | **Sample:** Control (first flush) | Bathroom faucet use (cold) | | | | |
|  |  | Bathroom faucet use (cold) |  |  |  |  |  |
| ~10:15 AM |  | **Sample:** Experiment (cold) After usage |  |  |  |  |  |
| 10:20 AM | Bathroom faucet use (mixed) | **Sample:** Control (first flush) | Bathroom faucet use (mixed) | | | | |
|  |  | Bathroom faucet use (mixed) |  |  |  |  |  |
| ~10:21 AM |  | **Sample:** Experiment (mixed) After usage |  |  |  |  |  |
| 10:30 AM | Kitchen faucet use (cold) | **Sample:** Control (first flush) | Kitchen faucet use (cold) | | | | |
|  |  | Kitchen faucet use (cold) |  |  |  |  |  |
| ~10:31 AM |  | **Sample:** Experiment (cold) After usage |  |  |  |  |  |
| 10:40 AM | Kitchen faucet use (mixed) | **Sample:** Control (first flush) | Kitchen faucet use (mixed) | | | | |
|  |  | Kitchen faucet use (mixed) |  |  |  |  |  |
| ~10:41 AM |  | **Sample:** Experiment (mixed) After usage |  |  |  |  |  |
| 1:30 PM | Kitchen faucet use (cold) | **Sample:** Control (first flush) | Kitchen faucet use (cold) | | | | |
|  |  | Kitchen faucet use (cold) |  |  |  |  |  |
| ~1:35 PM |  | **Sample:** Experiment (cold) After usage |  |  |  |  |  |
| 1:40 PM | Kitchen faucet use (mixed) | **Sample:** Control (first flush) | Kitchen faucet use (mixed) | | | | |
|  |  | Kitchen faucet use (mixed) |  |  |  |  |  |
| ~1:41 PM |  | **Sample:** Experiment (mixed) After usage |  |  |  |  |  |
| 1:50 PM | Bathroom faucet use (cold) | **Sample:** Control (first flush) | Bathroom faucet use (cold) | | | | |
|  |  | Bathroom faucet use (cold) |  |  |  |  |  |
| ~1:55 PM |  | **Sample:** Experiment (cold) After usage |  |  |  |  |  |
| 2:00 PM | Bathroom faucet use (mixed) | **Sample:** Control (first flush) | Bathroom faucet use (mixed) | | | | |
|  |  | Bathroom faucet use (mixed) |  |  |  |  |  |
| ~2:01 PM |  | **Sample:** Experiment (mixed) After usage |  |  |  |  |  |
| 2:10 PM | Bathroom faucet use (cold) | Bathroom faucet use (cold) | Bathroom faucet use (cold) | | | | |
|  |  |  |  |  |  |  |  |
| 2:20 PM | Bathroom faucet use (mixed) | Bathroom faucet use (mixed) | Bathroom faucet use (mixed) | | | | |
|  |  |  |  |  |  |  |  |
| 2:30 PM | Bathroom faucet use (cold) | Bathroom faucet use (cold) | Bathroom faucet use (cold) | | | | |
|  |  |  |  |  |  |  |  |
| 2:40 PM | Bathroom faucet use (mixed) | Bathroom faucet use (mixed) | Bathroom faucet use (mixed) | | | | |
|  |  |  |  |  |  |  |  |
| 2:50 PM | Bathroom faucet use (cold) | **Sample:** Control (first flush) | Bathroom faucet use (cold) | | | | |
|  |  | Bathroom faucet use (cold) |  |  |  |  |  |
| ~2:55 PM |  | **Sample:** Experiment (cold) After usage |  |  |  |  |  |
| 3:00 PM | Bathroom faucet use (mixed) | **Sample:** Control (first flush) | Bathroom faucet use (mixed) | | | | |
|  |  | Bathroom faucet use (mixed) |  |  |  |  |  |
| ~3:01 PM |  | **Sample:** Experiment (mixed) After usage |  |  |  |  |  |
| 6:30 PM | Kitchen faucet use (cold) | Kitchen faucet use (cold) | Kitchen faucet use (cold) | | | | |
|  |  |  |  |  |  |  |  |
| 6:40 PM | Kitchen faucet use (mixed) | Kitchen faucet use (mixed) | Kitchen faucet use (mixed) | | | | |
|  |  |  |  |  |  |  |  |
| 9:30 PM | Bathroom faucet use (cold) | Bathroom faucet use (cold) | Bathroom faucet use (cold) | | | | |
|  |  |  |  |  |  |  |  |
| 10:00 PM | Bathroom faucet use (mixed) | Bathroom faucet use (mixed) | Bathroom faucet use (mixed) | | | | |
|  |  |  |  |  |  |  |  |
| 10:10 PM | Bathroom faucet use (cold) | Bathroom faucet use (cold) | Bathroom faucet use (cold) | | | | |
|  |  |  |  |  |  |  |  |
| 11:00 PM | Bathroom faucet use (mixed) | Bathroom faucet use (mixed) | Bathroom faucet use (mixed) | | | | |
|  |  |  |  |  |  |  |  |
| 11:10 PM | Bathroom faucet use (cold) | Bathroom faucet use (cold) | Bathroom faucet use (cold) | | | | |
|  |  |  |  |  |  |  |  |
| 11:20 PM | Bathroom faucet use (mixed) | Bathroom faucet use (mixed) | Bathroom faucet use (mixed) | | | | |
|  |  |  |  |  |  |  |  |
| 11:30 PM | Bathroom faucet use (cold) | Bathroom faucet use (cold) | Bathroom faucet use (cold) | | | | |
|  |  |  |  |  |  |  |  |
| 11:40 PM | Bathroom faucet use (mixed) | Bathroom faucet use (mixed) | Bathroom faucet use (mixed) | | | | |
|  |  |  |  |  |  |  |  |
| 11:50 PM | Shower use (mixed) | Shower use (mixed) | Shower use (mixed) | | | | |
|  |  |  |  |  |  |  |  |

**Table S-4. Primers and probes for the Target OPPPs^a^**

| Target Species | Primer/Probe name | Primer/Probe Sequence | Reference: |
| --- | --- | --- | --- |
| *L. pneumophila* | mipF  mipR  mipP | 5’-AAAGGCATGCAAGACGCTATG-3’  5’- GAAACTTGTTAAGAACGTCTTTCATTTG-3’  5’-FAM- TGGCGCTCAATTGGCTTTAACCGA-BHQ1-3’ | Nazarian et al. (2008) |
| *N. fowleri* | itsF  itsR  itsP | 5’-GTGAAAACCTTTTTTCCATTTACA-3’  5’-AAATAAAAGATTGACCATTTGAAA-3’  5′-HEX-GTGGCCCACGACAGCTTT-BHQ1-3′ | Grimm et al. (2001) and Pélandakis et al. (2000) |
| *M. avium* | is1245F  is1245R  is1245P | F 5’-CGGGGAGTGGTGTAAGTGATG-3’  5’-CTCGGTAGTGATTCTTGGTCGTT-3’  5’-FAM-CCACAGAGACTCACGC-BHQ1 | Jiang et al. (2016) |
| *P. aeruginosa* | ecfxF  ecfxR  ecfxP | 5’-CGCATGCCTATCAGGCGTT-3’  5’-GAACTGCCCAGGTGCTTGC-3’  5’-HEX-ATGGCGAGTTGCTGCGCTTCCT-BHQ1 | Anuj et al., (2009) |

a: All primers and probes were designed and validated previously by the authors of the listed references

**References**

American Society of Heating, Refrigerating and Air-conditioning Engineers (ASHRAE), 2019. Chapter 51 Service Water Heating, in: 2019 ASHRAE® Handbook Heating, Ventilating, and Air-Conditioning Applications (S-I Edition). Atlanta, GA.

Anuj, S.N., Whiley, D.M., Kidd, T.J., Bell, S.C., Wainwright, C.E., Nissen, M.D., Sloots, T.P., 2009. Identification of Pseudomonas aeruginosa by a duplex real-time polymerase chain reaction assay targeting the ecfX and the gyrB genes. Diagnostic Microbiology and Infectious Disease 63, 127–131. https://doi.org/10.1016/j.diagmicrobio.2008.09.018

Grimm, D., Ludwig, W., Brandt, B., Michel, R., Schleifer, K.-H., Hacker, J., Steinert, M., 2001. Development of 18S rRNA-targeted Oligonucleotide Probes for Specific Detection of Hartmannella and Naegleria in Legionella – positive Environmental Samples. Systematic and Applied Microbiology 24, 76–82. https://doi.org/10.1078/0723-2020-00017

Helsel, D.R., 2012. Statistics for Censored Environmental Data Using Minitab® and R, 2nd ed. ed, Wiley series in statistics in practice. Wiley, Hoboken, N.J.

Jiang, Y., Ji, L., Wang, X., Li, G., Zhao, L., Dou, X., Wan, K., Lyu, J., 2016. Evaluation of a real-time PCR assay for detection of M. Avium strains. International Journal of Clinical and Experimental Pathology 9, 1487–1492.

Nazarian, E.J., Bopp, D.J., Saylors, A., Limberger, R.J., Musser, K.A., 2008. Design and implementation of a protocol for the detection of Legionella in clinical and environmental samples. Diagn Microbiol Infect Dis 62, 125–132. https://doi.org/10.1016/j.diagmicrobio.2008.05.004

Pélandakis, M., Serre, S., Pernin, P., 2000. Analysis of the 5.8S rRNA Gene and the Internal Transcribed Spacers in Naegleria spp. and in N. fowleri. Journal of Eukaryotic Microbiology 47, 116–121. https://doi.org/10.1111/j.1550-7408.2000.tb00020.x
